# Blood carotenoids as a biomarker of intestinal functionality and performance in broiler chickens

**DOI:** 10.3389/fphys.2025.1706730

**Published:** 2025-11-20

**Authors:** Letícia Cardoso Bittencourt, Robson Mateus Freitas Silveira, José Fernando Machado Menten

**Affiliations:** Department of Animal Science, “Luiz De Queiroz” College of Agriculture (ESALQ), University of São Paulo (USP), São Paulo, Brazil

**Keywords:** biomarkers, additives, intestinal functionality, performance, poultry

## Abstract

**Background:**

Research to establish a reliable biomarker that allows the practical and accurate evaluation of intestinal functionality and productive performance in broiler chickens is one of the main challenges in modern poultry production. In this context, blood carotenoids have stood out as promising indicators, as they reflect the absorptive efficiency and physiological balance of birds. The objective of this study was to verify whether the determination of total carotenoids in the blood can be used as an effective biomarker in experiments that evaluate nutritional additives, correlating them with zootechnical parameters.

**Materials and Methods:**

Data from five experiments were analyzed, totaling 9,125 broilers. Performance traits and blood total carotenoids were measured, and univariate and multivariate analyses were applied to evaluate the relationships between carotenoid status and performance.

**Results:**

Supplementation with nutritional additives resulted in a mean increase of 14% (*p* < 0.05) in blood carotenoid concentrations, regardless of the experimental design, diet, or the presence of a health challenge. A positive correlation was also observed between carotenoid levels and weight gain, and a negative correlation with feed conversion, during the periods from 0 to 35 and 0 to 42 days of age.

**Conclusion:**

The results indicate that the quantification of carotenoids in the blood, performed in a fast and minimally invasive way, can be used as an innovative tool to monitor intestinal functionality and predict productive performance in broilers in response to nutritional supplementation.

## Graphical Abstract

**Figure:**
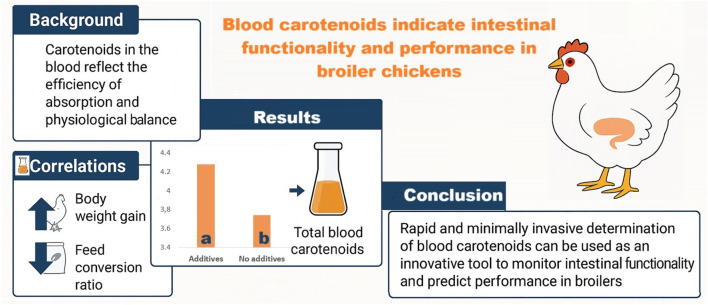

## Introduction

Carotenoids are fat-soluble pigments widely found in plants, microalgae, bacteria, and fungi, which cannot be synthesized by animals, so their presence in tissues or fluids is totally dependent on the diet (Miao et al., 2024). In poultry, carotenoids are responsible for the color of egg yolk, meat, and skin; in addition, these compounds have gained prominence for their antioxidant properties and potential role as health promoters (Nabi et al., 2020). Indeed, studies report that supplementing poultry diets with carotenoids can improve oxidative status, enhance immune responses, and ultimately boost growth performance and product quality (Nabi et al., 2020; Csernus et al., 2020).

Carotenoid absorption occurs in the small intestine and depends on the factors such as mucosal integrity, lipid micelle formation, and the efficiency of intracellular transport. The bioavailability can be modulated by dietary, genetic, and physiological aspects, including ingredient quality, fat, protein, and fiber levels; the presence of mycotoxins; and interactions with minerals and vitamins (Castenmiller and West, 1998). In this context, nutritional additives such as probiotics, prebiotics, enzymes, and plant extracts have been shown to be relevant for positively influencing the intestinal microbiota, reducing inflammation, and increasing digestive efficiency, favoring the absorption of fat-soluble compounds such as carotenoids (Shehata et al., 2022; Miao et al., 2023; Bittencourt and Menten, 2023).

A literature review conducted recently by Bittencourt and Menten (2023) presented evidence that the carotenoid absorption mechanism in chickens is associated with high digestive efficiency and thus to response in feed efficiency and growth. It was also shown that the degree of carotenoid absorption is measurable as carotenoid in peripheral blood. Intestinal damage or dysfunction can directly impact carotenoid status; for example, coccidiosis (*Eimeria* infection) in broilers significantly reduces plasma carotenoid levels, corresponding with gut lesions and depressed performance (Rochell et al., 2016). Mignon-Grasteau et al. (2020) observed that a chicken line selected for high feed efficiency had markedly greater serum carotenoid pigmentation than a low-efficiency line and showed a strong genetic correlation with digestive efficiency. Furthermore, when gut function is enhanced through dietary interventions, carotenoid levels respond accordingly.

Grounded in current evidence that dietary additives can modulate nutrient utilization and oxidative status in poultry, we propose that such supplementation will also be reflected in carotenoid status. Accordingly, our central hypothesis is that broilers receiving nutritional additives will exhibit higher circulating total carotenoid levels than unsupplemented birds and that blood carotenoid concentration will be positively associated with growth performance. Thus, in this study, we aimed (i) to compare blood total carotenoid levels between additive-supplemented and non-supplemented broilers and (ii) to quantify the relationship between blood carotenoids and standard performance metrics, evaluating whether blood carotenoids can serve as a practical biomarker in studies of nutritional additives and for predicting zootechnical outcomes.

## Materials and methods

Five prior experiments with broilers reared in floor pens were selected to evaluate the impact of nutritional additives on productive performance. Although aligned in purpose, the studies differed in commercial strains, facilities, treatment designs, diet types, and in whether they imposed nutritional challenges, microbiological challenges, or both. Four of the five experiments were conducted at the Animal Nutrition Center (ANC) of DSM Nutritional Products, Mairinque, São Paulo, Brazil (experiments 1–4), and one experiment was conducted at the Federal University of Paraná (UFPR), Curitiba, Paraná, Brazil (experiment 5). All procedures complied with Brazilian regulations for the ethical use of animals. Experiments 1–4 were approved by the DSM Ethics Committee on the Use of Animals (CEUA), and experiment 5 was approved by the UFPR CEUA.

Five experiments with broiler chickens were selected to evaluate the response of the biomarker in relation to productive performance. To ensure methodological comparability, only studies that quantified carotenoids in whole blood were included. Information on the year of conduction, the presence or absence of an experimental challenge, the number of litter-reuse cycles, and sample size is provided in Table 1; the ingredient composition and nutritional values of the grower diets used in each experiment are presented in Table 2. Diets of similar composition were supplied during the starter and finisher phases of each experiment; however, only the diets from the grower phase (21–35 days of age) are presented here because this was the period that included blood sampling and to avoid information irrelevant to the purpose of the present study. In each experiment, the feed additives were included at expense of the inert ingredient.

**TABLE 1:** Summary description of the assays that provided carotenoid blood level data. Whole blood tests of broilers at 28 and 35 days of age.

Trial	Year	Age (day)	Sex	Litter	Challenge	Sample N	Local
1	2016	28	Male	1st	Yes	96	ANC DSM^a^
2	2019	28	Male	1st	No	192	ANC DSM^a^
3	2021	28	Male	3 rd	No	198	ANC DSM^a^
4	2021	28	Male	5 th	Yes	85	ANC DSM^a^
5	2017	35	Male	3 rd	No	100	UFPR^b^

^a^ ANC DSM, *Animal Nutrition Center*, DSM Nutritional Products, Mairinque, SP.

^b^ UFPR, Federal University of Paraná, Curitiba, PR.

**TABLE 2:** Composition and nutritional levels of the diets used in the grower phase in the five experimental trials.

Ingredient, g kg^-1^ feed	Trial 1	Trial 2	Trial 3	Trial 4	Trial 5
Corn	557.06	640.6	651.2	637.8	601.8
Soybean meal	292.3	279.0	268.0	305.0	335.0
Meat and bone meal	26.4	28.0	35.0	—	—
Rice bran	60.0	—	—	—	—
Soybean oil	47.3	32.0	29.0	26.0	34.0
Dicalcium phosphate	—	—	—	7.7	7.5
Limestone	4.90	6.60	2.40	7.4	10.3
Salt	3.50	4.00	3.90	4.4	4.3
DL-methionine	2.80	2.90	2.80	2.95	2.50
L-lysine.HCl	2.10	2.55	2.55	2.50	0.61
L-threonine	0.500	1.00	1.00	1.10	—
Vitamin premix^a^	1.50	1.00	1.00	1.00	1.20
Mineral premix^b^	0.500	0.500	0.500	0.500	0.500
Phytase	0.100	0.050	0.050	0.050	0.100
Choline chloride 60%	—	0.50	0.50	0.60	0.60
Coccidiostat^c^	—	0.50	0.50	0.50	0.55
Apo-ester^d^	0.040	—	—	—	0.040
Inert	1.00	1.00	1.60	2.50	1.00
	1000	1000	1000	1000	1000
Nutritional level, g ^kg-1^ of feed					
MEn, kcal/kg	3200	3160	3150	3150	3100
Crude protein	200	203	200	190	200
Calcium	7.7	8.2	8.6	8.0	8.0
Phosphorus (available)	4.0	4.1	4.3	4.0	4.0
Digestible lysine	11.0	10.9	10.8	10.8	10.5
Digestible methionine	5.5	5.8	5.5	5.6	5.5
Digestible threonine	7.1	7.1	7.1	7.1	7.3
Digestible tryptophan	2.1	1.9	1.9	1.9	2.2

^a^ Vitamin premix: vitamin A 9,000,000 IU/kg; vitamin D_3_ 2,500,000 IU/kg; vitamin E 20,000 IU/kg; vitamin K_3_ 2,500 mg/kg; vitamin B_1_ 2,000 mg/kg; vitamin B_2_ 6,000 mg/kg; pantothenic acid 12 g/kg; vitamin B_6_ 3,000 mg/kg; vitamin B_12_ 15,000 mcg/kg; nicotinic acid 35 g/kg; folic acid 1,500 mg/kg; biotin 100 mg/kg; selenium 250 mg/kg of premix.

^b^ Mineral premix: iron 100 g/kg; copper 20 g/kg; manganese 130 g/kg; cobalt 2,000 mg/kg; zinc 130 mg/kg; iodine 2,000 mg/kg of premix.

^c^ RONOZYME® HiPhos GT, 20,000.

^d^ Salinomycin 12%, 66 ppm.

### Experimental trial 1

Eight hundred one-day-old Ross 308 broilers were allocated to four treatments, with eight replicates of 25 birds each. The birds were raised in new bedding, in an air-conditioned shed, for a period of 42 days, with feed and water *ad libitum*. In this study, different inclusions of the enzyme muramidase (0, 25,000, and 35,000 LSU/kg) and a positive control treatment with inclusion of 10 ppm of enramycin in the starter and grower phases and 5 ppm in the finisher phase were evaluated.

The diets were formulated according to industry levels, based on corn, soybean meal, rice bran, and meat and bone meal and with the inclusion of the phytase enzyme. Rice bran and meat and bone meal were included in the diets to create a nutritional challenge. In addition to the nutritional challenge, all birds were orally gavaged at 2 days of age with 15× the recommended dose of the Bio-Coccivet R coccidiosis vaccine (Biovet Laboratory®, *Eimeria acervulina*, *E. brunetti*, *E. maxima*, *E. necatrix*, *E. praecox*, *E. tenella*, and *E. mitis*, isolated from field strains), with the objective of provoking a challenge to the intestinal mucosa and simulating field situations. Blood samples were collected from broilers at 28 days of age, with 24 birds per treatment, totaling 96 samples, taken randomly and without prior fasting.

### Experimental trial 2

Two thousand four hundred one-day-old Cobb 500 broilers were allocated to eight treatments, each with 12 replicates of 25 birds. The animals were raised in new bedding, in an air-conditioned shed, for a period of 42 days, with feed and water *ad libitum*. In this study, different nutritional additives were evaluated: T1: blend of essential oils and organic acid; T2: T1 + muramidase; T3: T1 + protease; T4: T2 + protease; T5: enramycin; T6: T5 + muramidase; T7: T5 + protease; and T8: T6 + protease. The diets were formulated according to industry levels, based on corn and soybean meal and with the inclusion of the enzyme phytase.

Blood samples were collected from broilers at 28 days of age, with 24 birds per treatment, totaling 192 samples, taken randomly within each replication and without previous fasting.

### Experimental trial 3

Two thousand four hundred seventy-five one-day-old Cobb 500 broilers were allocated to nine treatments, each with 11 replicates of 25 birds. The animals were raised in an air-conditioned shed, placed on reused litter after two growing cycles, for a period of 42 days, with feed and water *ad libitum*. In this study, different nutritional additives were evaluated: T1: negative control treatment without additive (NC); T2: NC + muramidase; T3: NC + essential oil blend; T4: T3 + muramidase; T5: NC + essential oil blend and organic acid; T6: NC + organic acid; T7: NC + phytogenic; T8: NC + blend of organic acids; and T9: NC + prebiotic.

Blood samples were collected from broilers at 28 days of age, with 22 birds per treatment, totaling 198 samples, taken randomly within each replication and without prior fasting.

### Experimental trial 4

Two thousand two hundred one-day-old Cobb 500 broilers were allocated to eight treatments, each with 11 replicates of 25 birds. The animals were raised in an air-conditioned shed, placed on reused litter after four growing cycles, for a period of 42 days, with feed and water *ad libitum*. In this study, the microbiological challenge was evaluated as an additional factor. At 10, 11, and 12 days of age, the birds were orally gavaged with *Clostridium perfringens* (10^8^ CFU/bird—isolated from the field) to promote a challenge to the intestinal mucosa and simulate field situations.

Thus, the treatments were as follows: T1: treatment without challenge and without additive; T2: treatment with challenge and without additive; T3: T2 + enramycin; T4: T2 + blend 1 of essential oils and organic acid; T5: T2 + blend 2 of essential oils and organic acid; T6: T2 + prebiotic; T7: T2 + muramidase; and T8: T6 + muramidase. The diets were formulated according to industry levels, based on corn and soybean meal and with the inclusion of the enzyme phytase.

Blood samples were collected from broilers at 28 days of age, with 11 birds per treatment and 8 birds from the control group, totaling 85 samples, taken randomly within each replication and without previous fasting.

### Experimental trial 5

Two thousand two hundred one-day-old Cobb 500 broilers were allocated to eight treatments, each with 11 replicates of 25 birds. The animals were raised in reused litter for a period of 42 days, with feed and water *ad libitum*. In this study, different nutritional additives were evaluated: T1: negative control treatment without additive (NC); T2: positive control with enramycin; T3: NC + muramidase enzyme; T4: T3 + blend of essential oils with organic acid; and T5: T3 + phytogenic additive. The diets were formulated according to industry levels, based on corn and soybean meal (Table 2) and with the inclusion of phytase and xylanase enzymes. Blood samples were collected from broilers at 35 days of age, with 20 birds per treatment, totaling 100 samples, taken randomly and without prior fasting.

### Performance data collection

Feed intake was calculated as the difference between the feed offered and orts collected at the end of each experimental period, with values expressed as g/bird and also accumulated to the end of the trial. Birds were housed in experimental units with *ad libitum* access to water and feed under comparable conditions across treatments. Body weight gain (BWG) was determined by weighing at the start and end of each week (and cumulatively at trial end), performed individually or per pen, and expressed as g/bird or as cumulative gain. The feed conversion ratio (FCR) was computed as feed intake divided by weight gain over the same weekly interval, representing feed-use efficiency relative to growth. To avoid bias—particularly in intake and FCR—mortality was recorded daily, and performance calculations were adjusted for the number of birds in each unit.

### Collection and analysis of blood carotenoid levels

Blood sampling was performed at 28 days of age in experiments 1–4 and at 35 days in experiment 5. These trials were primarily designed to assess the effects of feed additives on broiler performance rather than carotenoid metabolism; therefore, complete harmonization of sampling age across all experiments was not feasible. To preserve biological comparability, sampling was intentionally scheduled after 21 days of age—that is, after the transition to the grower diet—when gastrointestinal functionality faces an additional challenge and circulating biomarkers stabilize under the new nutritional regime. This timing ensured that birds in all experiments were sampled under comparable post-transition physiological conditions, despite the 7-day difference in calendar age.

To determine the level of total carotenoids in the blood, blood from the brachial vein was collected into a tube containing EDTA, with a minimum volume of 1 mL to ensure proper homogenization with the anticoagulant. Determinations of blood total carotenoids were performed on the same day of collection, using iCheck™ CAROTENE equipment, a portable photometer from BioAnalyt (BioAnalyt GmbH, Teltow, Germany), which contains a carotenoid extraction kit for each sample (technical specifications in Table 3).

**TABLE 3:** Technical specifications of iCheck™ CAROTENE equipment and the iEX™ test kit.

Equipment	
Analyte	Total carotenoids
Method of analysis	Photometric determination of the total carotenoid concentration using absorption at 450 and 525 nm
Arrays	Blood, serum, and plasma
Sample volume	400 μL (0.4 mL)
Linear range	>0.15–15 mg/L
Accuracy in 95% confidence interval at 25 °C	±5%–20% (depends on sample type and concentration)
Method compared	Validated with HPLC (high-performance liquid chromatography)
Use	Portable
Analysis time	<10 min

Test kit	
Contains	Tubes with reagents (iEX™ extraction units), syringes, and needles (1.6 mm × 25 mm)
Chemical composition	N-hexane and alcohols
Volume per tube	2 mL
Validity	12 months at 20 °C–30 °C, no direct sunlight, vertical

A volume of 0.4 mL of blood was injected into the extraction unit, iEX™ tubes, followed by strong manual agitation for 10 s until a homogeneous mixture was obtained, with the blood cells completely mixed with the reagent. After homogenization, the tubes were left to stand for 5 min to allow carotenoid extraction and the separation of the tube contents into two phases. After separation, the iEx™ was placed into the equipment for instant reading, following the instructions provided in the equipment manual (iCheck™ CAROTENE User Manual/Version 10).

### Statistical analysis

As the experiments were conducted independently to evaluate a variety of unrelated feed additives as growth promoters, a summary of performance results illustrates the response obtained relative to the control diet (Table 4).

**TABLE 4:** Descriptive statistics of the variables under the study.

Experiment		FI 35d	BWG 35d	FCR 35 d	FI 42 d	BWG 42 d	FCR 42 d
1	Mean	4.09	2.60	1.57	5.53	3.24	1.71
	SEM	0.13	0.08	0.08	0.19	0.11	0.10
2	Mean	3.87	2.63	1.47	5.38	3.37	1.60
	SEM	0.09	0.07	0.04	0.15	0.11	0.04
3	Mean	3.89	2.70	1.44	5.25	3.32	1.58
	SEM	0.13	0.08	0.05	0.19	0.11	0.05
4	Mean	3.71	2.49	1.52	5.21	3.30	1.62
	SEM	0.21	0.07	0.06	0.24	0.09	0.06
5	Mean	3.18	2.04	1.56	4.38	2.61	1.68
	SEM	0.16	0.12	0.04	0.16	0.14	0.03
All	Mean	3.74	2.49	1.51	5.15	3.16	1.63
	SEM	0.35	0.27	0.08	0.44	0.31	0.08

BWG_35d and BWG_42d denote body weight gain accumulated over 0–35 and 0–42 days, respectively; FI_35d and FI_42d denote feed intake accumulated over the same intervals; FCR_35d and FCR_42d denote feed conversion ratio values for 0–35 and 0–42 days, respectively.

To evaluate the influence of the use of nutritional additives on carotenoid absorption, the results of the five experiments were submitted to joint data analysis, performed in the SAS PROC MIXED procedure at 5% probability, considering the experiment effect as random.

**FIGURE 1:**
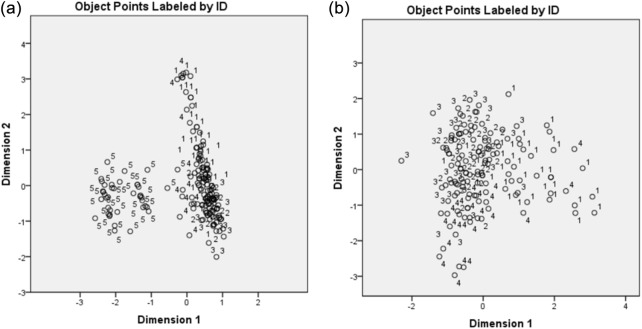
PCA score plots of performance (35 and 42 d) vs. carotenoids (28 d): **(a)** all five experiments; **(b)** four experiments (exp. 5 excluded). Note: axes show PC1 (dimension 1) and PC2 (dimension 2). Points are labeled by experiment; panel (2) illustrates the structure after removing experiment 5.

Principal component analysis (PCA) was used to examine the multivariate structure among variables and to assess potential between-experiment heterogeneity. All variables were mean-centered and standardized (z-scores), and the PCA was conducted on the correlation matrix. A two-step strategy was adopted: (i) an initial PCA using the pooled dataset from the five experiments to screen for location-related heterogeneity, and (ii) a prespecified sensitivity PCA excluding experiment 5—conducted at a different location—to minimize potential confounding by site. After this exclusion, the between-experiment effect was re-evaluated. Component retention followed conventional criteria (eigenvalues >1, scree plot inspection, and cumulative variance threshold), and the first two components—exceeding 70% cumulative variance—were retained for visualization. Scores and loadings were displayed in two-dimensional biplots to facilitate interpretation (Figures 1a,b).

The PCA was also performed to verify the relationship between performance variables and blood carotenoid levels. Subsequently, canonical discriminant analysis (CDA) was used to discriminate the main variables that differentiate the use of additives in broiler feed. The general model of CDA is described in Equation 1.

$$Z_{n}=\;\propto +\beta_{1}X_{1}+\beta_{2}X_{2}+\cdots +\beta_{n}X_{n},$$ (1)

where $Z_{n}$ is the dependent variable (with and without the use of additives), ∝ is the intercept, $X_{n}$ are the explanatory variables, and $\beta_{n}$ are the discriminant coefficients for each explanatory variable. The *stepwise* procedure was used to select independent variables with greater discriminatory power over dependent variables. This procedure is a data-mining tool that uses statistical significance to select the independent variables used in a given mathematical model (Smith, 2018). The selection process for the addition or removal of variables was carried out based on the Wilks Lambda statistical test (*p* < 0.05).

## Results and discussion

### Influence of nutritional additives on broiler performance and carotenoid absorption

Table 5 shows the blood levels of total carotenoids (mg/L) in broilers at 28 and 35 days of age, fed with or without the inclusion of nutritional additives in the diet. The joint analysis of the data shows that supplementation promoted a 14% increase (*p* < 0.05) in carotenoid absorption, regardless of the presence of a sanitary challenge. This result reinforces the potential of carotenoids as biomarkers of nutrient absorption efficiency, intestinal functionality, and, consequently, zootechnical performance.

**TABLE 5:** Blood levels of total carotenoids in broilers at 28 (experiments 1–4) or 35 days (experiment 5) of age with or without the addition of nutritional additive in the diet.

Nutritional additive	Blood level of total carotenoids (mg/L)
With	4.28a
Without	3.74b
Coefficient of variation, %	23.25
*p*-value	0.0013

Several authors highlighted that the quality of the ingredients and the composition of the diet directly influence the development and functionality of the gastrointestinal tract, being enhanced by the use of additives that modulate digestion, the microbiota, and the immune system (Celi et al., 2017; Idowu et al., 2025). In the present study, regardless of the type of the additive used, the increase in blood levels of carotenoids reflected an improvement in the gastrointestinal functionality of the birds.

This effect can be explained by the positive impact of additives on intestinal integrity and lipid metabolism. Carotenoid absorption, which occurs in the small intestine, depends on mucosal integrity, micelle formation, and intracellular transport (Nabi et al., 2020). Stressful situations or health challenges can compromise these steps, reducing the use of nutrients. Additives such as probiotics, prebiotics, enzymes, and plant extracts contribute to balancing the microbiota, reducing intestinal inflammation, and increasing lipid digestibility, favoring the absorption of fat-soluble compounds (Moreno et al., 2016; Zurak et al., 2024). In addition, they promote the production of short-chain fatty acids and the maintenance of villi, expanding the absorptive surface and ensuring greater use of nutrients (Bittencourt and Menten, 2023).

### Relationship of serum carotenoid levels with zootechnical performance

**FIGURE 2:**
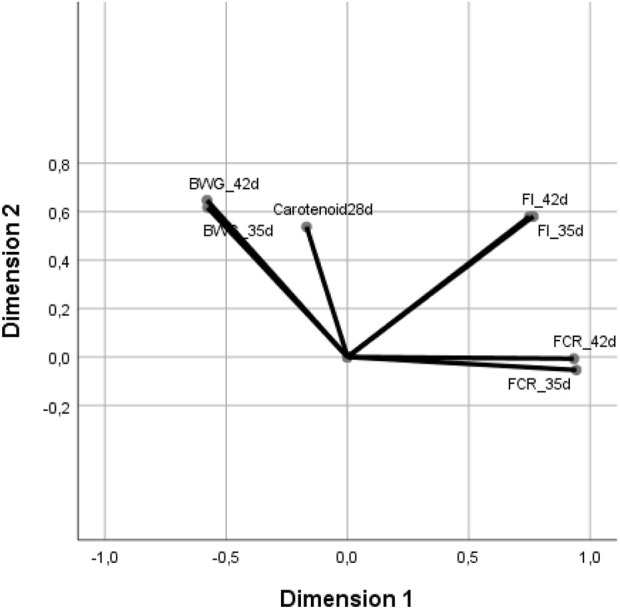
Relationship between performance in the periods 0–35 days and 0–42 days of age with blood carotenoid levels in broilers at 28 days of age. *Note:*BWG_35d and BWG_42d denote body weight gain accumulated over 0–35 and 0–42 days, respectively; FI_35d and FI_42d denote feed intake accumulated over the same intervals; FCR_35d and FCR_42d denote feed conversion ratio values for 0–35 and 0–42 days, respectively, and MCARO28 denotes total blood carotenoids measured at 28 days.

The positive correlation observed between blood carotenoid concentrations at 28 days and subsequent BWG (Table 2) aligns with previous research linking carotenoid status to broiler performance (Figure 2). Birds exhibiting higher plasma carotenoid levels tend to have better growth, which is consistent with the idea that carotenoid absorption reflects overall gut functionality and nutrient utilization (Bittencourt and Menten, 2023). These authors further stated that the plasma carotenoid level has been proposed as a robust biomarker of gastrointestinal functionality in broiler chickens, showing a consistent correlation with weight gain and feed efficiency. For example, Conway et al. (1993) demonstrated that even mild intestinal lesions from coccidial infection cause noticeable decreases in plasma carotenoid levels, with concomitant reductions in weight gain. Similarly, Beauclercq et al. (2019) reported that chickens genetically bred for high digestive efficiency had significantly greater serum carotenoid (lutein/zeaxanthin) levels than less efficient birds, indicating that better feed utilization is associated with higher carotenoid status. This explains the moderate negative relationship we found between carotenoids and FCR: birds with superior carotenoid absorption (and thus higher blood levels) converted feed more efficiently (lower FCR). Such a relationship is well supported by earlier studies showing that enhancing carotenoid availability or supplementation can improve poultry growth and reduce FCR (Yunitasari et al., 2023). Carotenoids’ antioxidant and anti-inflammatory roles may contribute directly to improved performance, but more importantly, a high carotenoid status indicates an intestine that is efficiently absorbing nutrients (Nabi et al., 2020). Therefore, the present findings—higher BWG and better FCR in birds with elevated plasma carotenoids—are in strong agreement with the literature: birds with greater carotenoid bioavailability tend to exhibit superior growth performance and feed efficiency (Bittencourt and Menten, 2023; Yunitasari et al., 2023).

The canonical discriminant analysis highlighted that feed intake (particularly over days 0–35 and 0–42; Table 6) and carotenoid levels at day 28 were the most influential variables distinguishing supplemented birds from unsupplemented birds. This finding suggests that dietary additives promoted higher feed consumption and nutrient uptake, which was reflected in elevated blood carotenoid concentrations. Such results are consistent with reports that certain feed additives (e.g., phytogenic compounds, probiotics, and enzymes) enhance gut function and appetite, thereby increasing both feed intake and the absorption of fat-soluble nutrients including carotenoids (Myers and Rochell, 2024). In a recent study, under *Eimeria* challenge, for instance, birds given a phytogenic additive showed improved feed intake and growth, along with signs of better intestinal health than challenged controls (Galamatis et al., 2025). Correspondingly, these interventions often lead to higher plasma carotenoid levels than those in non-supplemented, challenged birds, consistent with our observation. Thus, the superior classification success of the supplemented group (91.2% correct) in the discriminant function can be explained by the additives’ impact on these key parameters: they stimulated feed intake and helped sustain greater carotenoid bioavailability, ultimately translating to better growth performance. This interpretation is bolstered by prior findings that birds maintaining higher plasma carotenoid levels (due to improved nutrient absorption) exhibit more efficient nutrient utilization (Bittencourt and Menten, 2023). Overall, the close association of carotenoid status with feed efficiency and growth in our study is well founded in the existing literature, lending credence to the idea that carotenoid-rich blood profiles are indicative of optimally performing broilers.

**TABLE 6:** Summary of the canonical discriminant analysis for performance of broilers at 35 and 42 days and the carotenoid marker.

Variable in the analysis				
Step		Tolerance	F to remove	Wilks’ lambda
1	FI 42d	1.00	11.93	
2	FI 42d	0.20	22.03	0.98
	FI 35d	0.20	11.68	0.92
3	FI 42d	0.20	21.40	0.94
	FI 35d	0.20	11.57	0.89
	Carotenoids	1.00	5.33	0.85

Step	Exact F			
	Statistic	df1	df2	*p-*value
1	11.93	1.00	138.00	<0.001
2	12.27	2.00	137.00	<0.001
3	10.21	3.00	136.00	<0.001

Eigenvalue				
Function	Self-esteem	% of variance	Cumulative %	Canonical correlation
1	0.225	100.00	100.00	0.43

Standardized canonical discriminant function coefficient	
Variable	Function 1
Carotenoids	−0.45
Feed intake. 35d	−1.45
FI 42d	1.91

Classification of results			
		Predicted group membership	
		Yes	No
Additives	Yes	93.00 (91.18%)	9.00 (8.82%)
	No	24.00 (63.16%)	14.00 (36.84%)

FI, feed intake.

From a physiological point of view, this result can be understood because the supplemented birds had better intestinal functionality, as previously discussed; therefore, these animals exhibit preserved villi, less inflammation, and greater efficiency in absorbing fat-soluble compounds such as carotenoids. On the other hand, the birds in the control group possibly faced intestinal challenges, such as lesions in enterocytes or local inflammatory processes, which reduced the absorptive capacity and increased oxidative stress. Under these conditions, carotenoids may have been used primarily as antioxidants (Allen and Fetterer, 2002; Beauclercq et al., 2019), which explains its lower circulating levels. This intestinal imbalance, combined with the experimental conditions of challenge, contributes to the greater variability and worse performance observed in non-supplemented birds.

## Limitations

Blood carotenoid concentration represents a promising biomarker for assessing intestinal absorption in broilers, but their interpretation requires attention to certain influencing factors. Variations in feed intake, dietary lipid composition, body fat content, and the carotenoid concentration of feed ingredients—particularly corn—can affect circulating carotenoid levels, potentially masking true absorption efficiency. For example, corn from different regions or hybrids may contain markedly different levels of lutein and zeaxanthin, leading to fluctuations in baseline blood carotenoids unrelated to intestinal health. A strategy to mitigate this variability is the development of a standardized index that correlates dietary carotenoid intake with expected blood levels; such an index would help distinguish whether changes in blood carotenoid concentrations reflect intestinal absorptive capacity or merely differences in feed composition.

These factors do not invalidate the biomarker’s utility but highlight the importance of contextualizing results. By monitoring feed intake, dietary fat quality, ingredient variability, and body composition, research workers and nutritionists can enhance the accuracy of interpretations, making blood carotenoids a valuable tool in evaluating intestinal health when used with appropriate controls. Moreover, the continuation of large-scale evaluations will support the application of this tool beyond experimental settings, strengthening its relevance in commercial production environments.

## Conclusion

The determination of total carotenoid levels in the blood of broilers, carried out in a simple and practical way using portable equipment, represents a promising biomarker for experimental evaluations of nutritional additives. Regardless of the experimental design, the type of diet, or the presence of sanitary challenges, birds supplemented with additives aimed at improving intestinal functionality showed significantly higher blood concentrations of carotenoids. In addition, a significant correlation was observed between these levels and zootechnical performance indicators, such as weight gain and feed conversion, reinforcing the association between intestinal health and efficiency in the use of nutrients. Nevertheless, in the absence of confirmatory pathological examination and mechanistic assessments of small-intestinal integrity (e.g., histopathology, villus–crypt morphometry, tight-junction protein expression, permeability tests, and inflammatory markers), these associations should be interpreted cautiously; targeted studies integrating pathology, functional assays, and performance endpoints are required to substantiate causality and consolidate the use of blood carotenoids as a biomarker.

These findings highlight the potential of carotenoid measurement as a practical and innovative tool for monitoring intestinal functionality and for developing more efficient and sustainable nutritional strategies in poultry production.

## Data Availability

The raw data supporting the conclusions of this article will be made available by the authors, without undue reservation.

## References

[B2] Allen P.C. Fetterer R.H. (2002). Interaction of dietary vitamin E with *Eimeria maxima* infections in chickens. Poult. Sci. 81, 41–48. 10.1093/ps/81.1.41 11885898

[B3] Beauclercq S. Nadal-Desbarats L. Germain K. Praud C. Emond P. Bihan-Duval E.L. (2019). Does lipidomic serum analysis support the assessment of digestive efficiency in chickens? Poult. Sci. 98, 1425–1431. 10.3382/ps/pey483 30325459 PMC6377433

[B4] Bittencourt L.C. Menten J.F. M. (2023). Carotenoids blood level as biomarker for intestinal functionality in broiler chicken trials: a review. Asian J. Anim. Vet. Adv. 18, 108–112. 10.3923/ajava.2023.108.112

[B6] Castenmiller J.J. West C. (1998). Bioavailability and bioconversion of carotenoids. Annu. Rev. Nutr. 18, 19–38. 10.1146/annurev.nutr.18.1.19 9706217

[B7] Celi P. Cowieson A.J. Fru-Nji F. Steinert R.E. Kluenter A.M. Verlhac V. (2017). Gastrointestinal functionality in animal nutrition and health: new opportunities for sustainable animal production. Anim. Feed Sci. Technol. 234, 88–100. 10.1016/j.anifeedsci.2017.09.012

[B8] Conway D.P. Sasai K.S. Gaafar M. Smothers C.D. (1993). Effects of different levels of oocyst inocula of *Eimeria acervulina, E. tenella* and *E. maxima* on plasma constituents, packed cell volume, lesion scores and performance in chickens. Avian Dis. 37, 118–123. 10.2307/1591464 8452488

[B25] Csernus B. Biró S. Babinszky L. Komlósi I. Jávor A. Stündl L. (2020). Effect of carotenoids, oligosaccharides and anthocyanins on growth performance, immunological parameters and intestinal morphology in broiler chickens challenged with Escherichia coli lipopolysaccharide. Animals, 10 (2), 347. 10.3390/ani10020347 32098265 PMC7070938

[B10] Galamatis D. Stylianaki I. Mantzios T. Makri V. Papadopoulos E. Caldwell J. (2025). The effectiveness of phytobiotic additives on performance and intestinal health of broilers after challenging with *Eimeria* spp. Discov. Anim. 2, 32. 10.1007/s44338-025-00074-x

[B11] Idowu P.A. Mpofu T.J. Magoro A.M. Modiba M.C. Nephawe K.A. Mtileni B. (2025). Impact of probiotics on chicken gut microbiota, immunity, behavior, and productive performance—a systematic review. Front. Anim. Sci. 6, 1562527. 10.3389/fanim.2025.1562527

[B12] Miao Q. Tang C. Yang Y. Zhao Q. Li F. Qin Y. (2023). Deposition and bioconversion law of β-carotene in laying hens after long-term supplementation under adequate vitamin A status in the diet. Poult. Sci. 102, 103046. 10.1016/j.psj.2023.103046 37708765 PMC10502406

[B13] Miao Q. Si X. Zhao Q. Zhang H. Qin Y. Tang C. (2024). Deposition and enrichment of carotenoids in livestock products: an overview. Food Chem. X 21, 101245. 10.1016/j.fochx.2024.101245 38426078 PMC10901861

[B14] Mignon-Grasteau S. Beauclercq S. Urvoix S. Bihan-Duval E. L. (2020). Interest in the serum color as an indirect criterion of selection of digestive efficiency in chickens. Poult. Sci. 99, 702–707. 10.1016/j.psj.2019.10.005 32036974 PMC7587746

[B15] Moreno J. Díaz-Gómez J. Nogareda C. Angulo E. Sandmann G. Portero-Otin M. (2016). The distribution of carotenoids in hens fed on biofortified maize is influenced by feed composition, absorption, resource allocation and storage. Sci. Rep. 6, 35346. 10.1038/srep35346 27739479 PMC5064355

[B16] Myers A. G. Rochell S. J. (2024). Effects of starter diet energy concentration on nutrient digestibility and subsequent growth performance and meat yields of broilers under two coccidiosis control programs. Anim. (Basel) 14 (11), 1524. 10.3390/ani14111524 38891571 PMC11171360

[B17] Nabi F. Arain M.A. Rajput N. Alagawany M. Soomro J. Umer M. (2020). Health benefits of carotenoids and potential application in poultry industry: a review. J. Anim. Physiol. Anim. Nutr. 104, 1809–1818. 10.1111/jpn.13375 32333620

[B19] Rochell S. J. Parsons C. M. Dilger R. N. (2016). Effects of *Eimeria acervulina* infection severity on growth performance, apparent ileal amino acid digestibility, and plasma concentrations of amino acids, carotenoids, and α1-acid glycoprotein in broilers. Poult. Sci. 95 (7), 1573–1581. 10.3382/ps/pew035 26933234

[B20] Shehata A. A. Yalçın S. Latorre J. D. Basiouni S. Attia Y. A. Abd El-Wahab A. (2022). Probiotics, prebiotics, and phytogenic substances for optimizing gut health in poultry. Microorganisms 10, 395. 10.3390/microorganisms10020395 35208851 PMC8877156

[B21] Smith G. (2018). Step away from stepwise. J. Big Data 5, 32. 10.1186/s40537-018-0143-6

[B23] Yunitasari F. Jayanegara A. Ulupi N. (2023). Performance, egg quality, and immunity of laying hens due to natural carotenoid supplementation: a meta-analysis. Food Sci. Anim. Resour. 43 (2), 282–304. 10.5851/kosfa.2022.e76 36909857 PMC9998190

[B24] Zurak D. Svečnjak Z. Gunjević V. Kiš G. Janječić Z. Pirgozliev V. (2024). Carotenoid content and deposition efficiency in yolks of laying hens fed with dent corn hybrids differing in grain hardness and processing. Poult. Sci. 103, 103750. 10.1016/j.psj.2024.103750 38652952 PMC11063521

