# Correction: Blood carotenoids as a biomarker of intestinal functionality and performance in broiler chickens

**DOI:** 10.3389/fphys.2026.1788078

**Published:** 2026-01-22

**Authors:** Letícia Cardoso Bittencourt, Robson Mateus Freitas Silveira, José Fernando Machado Menten

**Affiliations:** Department of Animal Science, “Luiz De Queiroz” College of Agriculture (ESALQ), University of São Paulo (USP), São Paulo, Brazil

**Keywords:** biomarkers, additives, intestinal functionality, performance, poultry

In the published article, we inadvertently omitted the AI Use Disclosure. We apologize for this omission. The **Generative AI statement** should be:

The author(s) declared that generative AI was used in the creation of this manuscript. ChatGPT was used during the preparation of this manuscript to support language refinement, readability, and the preparation of the graphical abstract. All scientific content, data interpretation, and final decisions were made by the authors, who thoroughly reviewed and edited the text and take full responsibility for the manuscript.

Any alternative text (alt text) provided alongside figures in this article has been generated by Frontiers with the support of artificial intelligence and reasonable efforts have been made to ensure accuracy, including review by the authors wherever possible. If you identify any issues, please contact us.

The original article has been updated.

